# Perspectives of Patients With Chronic Respiratory Diseases and Medical Professionals on Pulmonary Rehabilitation in Pune, India: Qualitative Analysis

**DOI:** 10.2196/45624

**Published:** 2023-11-07

**Authors:** Rashmi Padhye, Shruti D Sahasrabudhe, Mark W Orme, Ilaria Pina, Dipali Dhamdhere, Suryakant Borade, Meenakshi Bhakare, Zahira Ahmed, Andy Barton, Mahavir Modi, Dominic Malcolm, Sundeep Salvi, Sally J Singh

**Affiliations:** 1 Clinical Research Department Symbiosis Medical College for Women Symbiosis University Hospitals and Research Centre, Symbiosis (Deemed University) Pune India; 2 Department of Respiratory Sciences University of Leicester Leicester United Kingdom; 3 Centre for Exercise and Rehabilitation Science NIHR Leicester Biomedical Research Centre-Respiratory University Hospitals of Leicester NHS Trust Leicester United Kingdom; 4 Department of Respiratory Medicine Symbiosis Medical College for Women Symbiosis (Deemed University) Pune India; 5 Pulmonology Department Ruby Hall Clinic Pune India; 6 School of Sport, Exercise and Health Sciences Loughborough University Loughborough United Kingdom; 7 Pulmonary Research and Education Foundation Pune India

**Keywords:** COPD, chronic obstructive pulmonary disease, asthma, patients’ suffering, self-management, digital mode of PR, integrating yoga with PR, thematic qualitative analysis, knowledge about PR, barriers to PR, chronic respiratory diseases, CRD, India, pulmonary rehabilitation, medical professional, treatment, inhaler

## Abstract

**Background:**

Chronic respiratory diseases (CRDs) contribute significantly to morbidity and mortality worldwide and in India. Access to nonpharmacological options, such as pulmonary rehabilitation (PR), are, however, limited. Given the difference between need and availability, exploring PR, specifically remotely delivered PR, in a resource-poor setting, will help inform future work.

**Objective:**

This study explored the perceptions, experiences, needs, and challenges of patients with CRDs and the potential of and the need for PR from the perspective of patients as well as medical professionals involved in the referral (doctors) and delivery (physiotherapists) of PR.

**Methods:**

In-depth qualitative semistructured interviews were conducted among 20 individuals diagnosed with CRDs and 9 medical professionals. An inductive thematic analysis approach was used as we sought to identify the meanings shared both within and across the 2 participant groups.

**Results:**

The 20 patients considered lifestyle choices (smoking and drinking), a lack of physical activity, mental stress, and heredity as the triggering factors for their CRDs. All of them equated the disease with breathlessness and a lack of physical strength, consulting multiple doctors about their physical symptoms. The most commonly cited treatment choice was an inhaler. Most of them believed that yoga and exercise are good self-management strategies, and some were performing yoga postures and breathing exercises, as advised by friends or family members or learned from a televised program or YouTube videos. None of them identified with the term “pulmonary rehabilitation,” but many were aware of the exercise component and its benefits. Despite being naive to smartphone technology or having difficulty in reading, most of them were enthusiastic about enrolling in an application-based remotely delivered digital PR program. The 9 medical professionals were, however, reluctant to depend on a PR program delivered entirely online. They recommended that patients with CRDs be supported by their family to use technology, with some time spent with a medical professional during the program.

**Conclusions:**

Patients with CRDs in India currently manage their disease with nonguided strategies but are eager to improve and would benefit from a guided PR program to feel better. A home-based PR program, with delivery facilitated by digital solutions, would be welcomed by patients and health care professionals involved in their care, as it would reduce the need for travel, specialist equipment, and setup. However, low digital literacy, low resource availability, and a lack of expertise are of concern to health care professionals. For India, including yoga could be a way of making PR “culturally congruent” and more successful. The digital PR intervention should be flexible to individual patient needs and should be complemented with physical sessions and a feedback mechanism for both practitioners as well as patients for better uptake and adherence.

## Introduction

Chronic respiratory diseases (CRDs), including chronic obstructive pulmonary disease (COPD) and asthma, contribute significantly to morbidity and mortality worldwide. India has experienced a huge surge in COPD cases (by 27.2 million from 1990 to 2016) [1]. A recent systematic review [2] estimated a COPD prevalence of 7.4% (from 8 identified studies with a pooled sample of 8569 individuals) among adults in India. India accounts for 32% of the total worldwide disability-adjusted life years (DALYs) due to CRDs [1]. Experiences of breathlessness [3], chronic cough [4], and exercise intolerance [5] are common across CRDs, limiting patients’ ability to perform routine activities. At the worldwide level, awareness about CRDs, their prevention, and their management is low and in disproportion to the prevalence [6]. Patients with CRDs often rely on treatments that offer quick, short-term relief from these physical symptoms, typically pharmacological treatments [7]. Access to nonpharmacological options, such as pulmonary rehabilitation (PR), are limited worldwide [8] and in India [9].

PR is a “comprehensive, structured, assessment-based and patient-tailored, exercise and education-based intervention that relies on behavior change and long term adherence for improvement in physical and psychological condition” of patients with CRDs [10]. PR has proved to be crucial in improving patients’ quality of life by reducing suffering, relieving anxiety [11], and increasing exercise capacity [12]. Despite unequivocal evidence for the health benefits of PR [13], its availability worldwide remains low. Evidence of the effectiveness of PR in low- and middle-income countries (LMICs) is limited [14]; however, a few examples highlight the barriers in these low-resource settings. A qualitative study [14] among medical professionals across several LMICs documented limited resource availability, including a lack of professionals with proper knowledge about PR and a lack of equipment. The study [14] further listed a lack of recognition of CRDs, a lack of knowledge of the treatment, and the direct and indirect costs of the treatment as patient-level barriers. A study in Uganda [15] highlighted the need for a tailored training program for the referrers to aid the success of a PR program for patients with CRDs, including COPD and posttuberculosis lung disease (PTLD). Similarly, a study in Sri Lanka [16] recommended an education program for the referrers for effective implementation of PR for patients with COPD.

Other barriers include issues in transportation for patients and difficulties in following the strict schedule of the PR program. Difficulties in travel and transport are cited by many patients. Comorbidities and disabilities, and disruption of their routines to attend in-person PR, have been reported as major concerns [17,18]. These factors indicate the need for alternative modes that can increase the capacity to deliver PR and contribute to offering the patient a suite of options to help tailor PR to their individual circumstances. One such example is digital home-based PR, delivered remotely by a trained workforce, which can help increase PR accessibility and use [9,19]. During the SARS-CoV-2 (COVID-19) pandemic, remote PR became the only way for health care providers to support patients, making the concept better accepted by patients and families [20].

PR is still not part of routine clinical practice in India. PR, in its nascent state, is limited to clinical trials. A few medical professionals, especially in metropolitan cities, are offering PR to patients with COPD, along with pharmacological treatment [21]. Given the scale of the problem in India and the lack of PR programs that cater to the increasing need, exploring not only PR but also specifically PR delivered digitally and at home will help inform how to implement appropriate PR programs in India.

In this study, we explored the perceptions, experiences, needs, and challenges of individuals with 2 of the most common CRDs, COPD and chronic asthma, and the potential of and the need for PR from the perspective of patients as well as medical professionals involved in the referral (doctors) and delivery (physiotherapists) of PR.

## Methods

### Study Design

A qualitative study was conducted between May and December 2021 under the National Institute for Health Research (NIHR) Global RECHARGE project [22]. Semistructured qualitative interviews were conducted with patients with CRDs and the medical professionals treating patients with CRDs.

### Participants

Patients with chronic asthma and COPD and medical professionals (doctors and physiotherapists) experienced in treating patients with CRDs residing in Pune City, India, were invited to participate in the study. Patients with CRDs were chosen using a convenience sampling method [23]. This included inviting patients with CRDs seeking treatment at a private multispecialty hospital and a private chest clinic. The inclusion criteria for patients with CRDs were a confirmed diagnosis of CRD by a physician, age≥18 years, and a willingness to participate in the study. For medical professionals, snowball sampling was used, whereby those interviewed were asked to share contact details of other potentially suitable participants [24]. The research team approached the medical professionals based on these referrals. The objectives and scope of the study were explained over a phone call, and participants were asked to make an appointment for the interview. Those medical professionals who responded to these calls and made appointments were interviewed. The average response time for scheduling an interview was 10 days.

### Data Collection

In-depth qualitative semistructured interviews were conducted with 20 patients with CRDs (n=13, 65%, COPD and n=7, 35%, chronic asthma) and 9 medical professionals (n=4, 44.4%, doctors and n=5, 55.6%, physiotherapists). Given the procedures of wearing personal protective equipment (PPE) and following social distancing and the risk of contracting SARS-CoV-2 while traveling or in the hospital setting, 5 (25%) patients with CRDs and 2 (22.2%) medical professionals who did not wish to attend in person were interviewed on WhatsApp video calls.

The patient interview guide (see Multimedia Appendix 1) included questions about their experiences of living with CRDs, ways of managing the condition, their daily routine and exercises, and their perspectives on PR and specifically potential digital modes of PR. The interviews with medical professionals (see Multimedia Appendix 2 for the interview guide) focused on their experiences of treating patients with CRDs, their views and experiences of PR, their perspectives on different models of PR that may be suitable for the local setting, and their views on anticipated patient responses to these models (including the digital mode of PR).

The interviews were conducted in Marathi, the regional language of Maharashtra, the state in which the study was conducted (n=20, 100%, patients and n=0 medical professionals); Hindi, the official language of India (n=0 patients and n=1, 11.1%, medical professional); or a mixture of Marathi and English (n=0 patients and n=8, 88.9%, medical professionals), depending on participant preference. The interviewer (author RP) was fluent in all 3 languages. The mean interview duration was 30 (range 19-60) minutes. All the interviews were conducted by RP. Authors DD and SDS were present as an observer and a note taker, respectively, for patient interviews. SDS was also present during the interviews of the medical professionals, assisting RP in understanding some of the medical terminology and the context of the responses. The interviews were audio-recorded with the participants’ consent. The recorded interviews were then transcribed verbatim and translated into English.

### Data Analysis

An inductive thematic analysis approach was used as we sought to identify the meanings shared both within and across the 2 participant groups. Data analysis was performed using the 6 steps of thematic analysis [25], repeating steps 1-3 several times. The details of the analysis are given in Table 1. The data of the patients and medical professionals were analyzed together.

**Table 1:** Inductive thematic analysis process used in the study.

Steps	Description of the process
Step 1: Data familiarization	Once the transcripts were ready, the team members (authors RP, SDS, MWO, and IP) read and reread the interviews to become familiar with the data. Some ideas for the codes were noted at this point.
Step 2: Preliminary coding	RP coded all the transcripts and prepared a free list of codes. SDS, MWO, and IP each independently coded a subset of transcripts. Resulting codes were discussed as a team via video calls.
Step 3: Collating the codes with the help of supporting quotes from the interviews	RP refined and grouped the codes into potential themes and subthemes. These were discussed with SDS (face to face/video call) and MWO (video call), independently and together. Based on the discussions, some ideas for grouping the codes to form themes and subthemes were generated.
Step 4: Grouping the codes according to themes and subthemes	RP then grouped the codes based on the discussions into fully drafted themes and subthemes.
Step 5: Review and merging of the themes	The themes were reviewed (authors RP, SDS, MWO, DM), duplicates were removed, and themes were merged to form a final set of themes.
Step 6: Summary of each theme with relevant quotes	RP then summarized all the themes and subthemes to complete the “Results” section of the manuscript. Quotes from the participants were used to enrich and explain each of the themes and subthemes. All team members provided input for the finalization of themes. The final themes and quotes were reviewed and approved by all authors.

### Ethical Considerations

Ethical approval was obtained from the Independent Ethics Committee of Symbiosis International (Deemed) University (ref: SIU/IEC/218) and the Medicine and Biological Sciences Research Ethics Committee of the University of Leicester (ref: 22819). All participants provided written informed consent prior to data collection. The consent form was read out to participants who could not read, and their thumb impressions were taken prior to the interviews. Each interviewee was deidentified (using patient IDs and doctor/physiotherapist IDs) immediately after transcription, only keeping the age, sex, and type of CRD in the patients’ description and the sex in the medical professionals’ description.

## Results

### Patients’ Characteristics

Table 2 gives a brief description of the demographic characteristics of patients.

**Table 2:** Individual patient (N=20) description.

Patient number	Sex	Age (years)	Education level	CRD^a^ diagnosis	Years since diagnosis
1	Male	64	Higher secondary	COPD^b^	10
2	Male	79	No education	COPD	14
3	Male	61	No education	COPD	7
4	Male	79	Secondary	COPD	7
5	Male	79	Higher secondary	Asthma	13
6	Female	71	No education	Asthma	6
7	Female	43	Higher secondary	Asthma	10
8	Female	69	Secondary	Asthma	2
9	Male	58	Primary	COPD	2
10	Male	69	Higher secondary	COPD	2
11	Male	48	Higher secondary	COPD	15
12	Female	44	Secondary	COPD	2
13	Male	61	Secondary	COPD	30
14	Male	67	Secondary	COPD	10
15	Female	52	Primary	COPD	25
16	Female	56	Secondary	Asthma	30
17	Male	77	Higher secondary	COPD	1
18	Male	65	Higher secondary	COPD	20
19	Female	71	Higher secondary	Asthma	7
20	Male	53	Higher secondary	Asthma	15

^a^CRD: chronic respiratory disease.

^b^COPD: chronic obstructive pulmonary disease.

The patients’ median age was 65 (range 43-79) years. Of the 20 patients, 11 (55%) resided in urban areas around Pune and 8 (40%, all male) had smoked cigarettes previously but none confirmed to have a current smoking habit. In addition, 2 (10%) were currently being exposed to the use of biomass fuel in the household and 12 (60%) had been exposed to it in the past. Furthermore, 8 (40%) patients reported working at the time of the interview (n=3, 37.5%, working on their farms; n=3, 37.5%, salaried employees; and 2, 25%, self-employed).

### Medical Professionals’ Characteristics

Medical professionals interviewed comprised 4 (44.4%) doctors with an advanced degree in pulmonology (n=2, 50%, males and n=2, 50%, females) and 5 (55.6%) physiotherapists with the knowledge of PR (n=3, 60%, males and n=2, 40%, females). In addition, 6 (66.7%) medical professionals had private clinical setups, whereas 2 (22.7%) doctors and 1 (11.1%) physiotherapist were working in a private multispecialty hospital. All the doctors had more than 20 years of experience, and all the physiotherapists had more than 10 years of experience.

### Themes and Subthemes

The themes and subthemes identified from the data are summarized in Table 3. Theme 1 was drawn only from the responses of the patients, whereas theme 2 was drawn from the responses of both patients and medical professionals.

**Table 3:** Themes and subthemes used for the analysis.

Themes and subthemes		Description
**Theme 1: Living with CRDs^a^**		
	Subtheme 1.1	Perceptions about causation
	Subtheme 1.2	Patients’ suffering
	Subtheme 1.3	Management strategies
**Theme 2: Perceptions of PR^b^**		
	Subtheme 2.1	Knowledge of PR
	Subtheme 2.2	Barriers to PR
	Subtheme 2.3	Digital mode of PR

^a^CRD: chronic respiratory disease.

^b^PR: pulmonary rehabilitation.

#### Theme 1: Living With CRDs

The first theme highlighted the patients’ perceptions and experiences of CRDs and their management strategies.

##### Subtheme 1.1: Perceptions of Causation

All patients expressed their views about the etiology of their CRD. The most common belief among men was lifestyle choices, such as smoking and drinking, while women linked their condition to a lack of physical activity and mental stress.

I smoked in the past, I also used to consume alcohol. This (breathlessness) could be a result of that.

Patient 2, male, 70 years old, COPD

I used to be a worker at a goldsmith’s factory previously. I used to do the work of blowing over the furnace for 30 years. Since last 10 years, I have stopped that work. Back then, I also used to smoke bidis (a hand-rolled cigarette) a lot.

Patient 14, male, 67 years old, COPD

I used to work in paddy fields. Continuously being in water gave me cough, cold more often. I stopped working on the farms a few years back. Later, I started feeling breathless. Now, the doctors tell me that my body is not getting any exercise because of which I’m facing so much trouble.

Patient 8, female, 69 years old, asthma

My husband met with an accident and was paralyzed. My sons, who were studying at other places, came home. Mother-in-law is with me. Between the household work, husband’s exercise, and all this, there is tension. Breathlessness started once again with this.

Patient 7, female, 43 years old, asthma

Some of the patients who only started to experience chronic symptoms after their retirement or during the COVID-19 pandemic believed that physical inactivity caused the condition.

This (breathing trouble) started because I was bound to home. When I was working, it was all well.

Patient 1, male, 64 years old, COPD

Heredity was also considered a triggering factor for CRDs.

I am suffering since the last 25 years. It’s hereditary. My grandfather and father had it. Even my son has it.

Patient 11, male, 48 years old, COPD

##### Subtheme 1.2: Patients’ Suffering

While talking about the why and when of treatment, patients discussed at length the internal cues for treatment, namely the suffering they experienced because of their condition. All patients suffered from breathlessness and equated the disease with it. Most patients talked about how they suffered from breathlessness year-round but experienced fluctuations based on the weather, worsening during cold or humid weather. One person described their body’s response to weather changes as:

In cloudy weather, I become breathless. I get very restless. I feel as if I’m dying.

Patient 8, female, 69 years old, asthma

Other sufferings were quoted as:

My sleep is disturbed, and I cough after every half an hour.

Patient 9, male, 58 years old, COPD

Wheezing sound while I sleep is very loud. It disturbs the family members, even in the next room.

Patient 15, female, 52 years old, COPD

Patients expressed helplessness due to their CRDs and the physical inability to do any kind of work.

I wake up in the morning, pray to God, then I have my breakfast and sit. No exercise. No work. I don’t have strength to perform exercise. I have ridden a bicycle for 36 years. And now this.

Patient 13, male, 61 years old, COPD

Physical limitations led to a partial dependence on family members (eg, to get a bucketful of water for bathing or to get dressed). One of the respondents mentioned:

I can’t pick up anything heavy. If I try to do that, I need to sit immediately. I also can’t kick-start my vehicle.

Patient 1, male, 64 years old, COPD

The patients came up with their own adjustments to perform their routine activities. Their solutions included:

I still do the daily household chores. I get breathless while walking, while climbing the stairs. I just sit at one place then. And continue the work afterwards.

Patient 19, female, 71 years old, asthma

I work in housekeeping. I go to work. I keep my inhaler in my pocket. If I feel breathless while on duty, I go to the washroom, use my inhaler, sit there for 5-10 minutes, and resume work.

Patient 15, female, 52 years old, COPD

##### Subtheme 1.3: Management Strategies

All patients had consulted multiple medical professionals about their physical symptoms. The health facilities explored by the patients ranged from general practitioners working in villages or urban settlements to public health facilities near their place of residence, respiratory medicine specialists, and multispecialty hospitals, depending on the severity of symptoms. The visit to a multispecialty hospital happened in almost all cases on the recommendation of a doctor, relative, or friend. Some of the management strategy–related responses included:

The doctors started with the inhalers. There were tablets too. The medicines were quite effective as well. I used to feel relieved.

Patient 3, male, 61 years old, COPD

I took medicines from a military hospital. They gave me an inhaler too. I did not use it though. It stopped with medicines. Now, there is 1 private clinic near my residence in the village. I visit it for breathlessness every now and then. I feel okay.

Patient 6, female, 71 years old, asthma

The patients sought care from all allied and locally rooted medicine branches, such as allopathy and ayurveda, as these are more relatable. They also came up with their own simple lifestyle changes for symptomatic relief and exercised and practiced yoga based on suggestions by family and friends.

For my condition, I have tried ayurvedic and homeopathic treatment. Experts (from among acquaintances) have suggested me to use drops, and I do exercises also suggested by my seniors in the company and the doctors.

Patient 11, male, 48 years old, COPD

The next sections discuss these management strategies in further detail.

### Pharmacotherapy

The most commonly cited treatment choice for the patients was an inhaler. The use of inhalers ranged from occasionally, whenever they felt breathless, to several times during the day and even between their routine activities. For instance, a female patient said:

I have to take the inhaler 7 times a day. After using the inhaler for so many times also, at times it (breathlessness) is not under control.

Patient 7, female, 43 years old, asthma

For some, using the inhaler is the first thing they do upon waking up. For most, because of their dependence on the inhaler, they carry it everywhere and use it often. One female respondent with no education shared her challenges while using an inhaler by stating:

They have given me the inhaler, but I don’t understand how to use it. If I use just the inhaler, I can, but through the pipe, it becomes difficult. The medicine fumes don’t come.

Patient 6, female, 71 years old, asthma

Other treatment options for symptomatic relief included tablets in the case of exacerbation (reported by all patients) and a cough syrup. The patients had preferred combinations of medicines, and some of them mentioned purchasing these over the counter.

I was afraid to go to the hospital because they changed the medicines many times. Instead, I went to the medical store and purchased medicines.

Patient 4, male, 79 years old, COPD

### Self-Management

Most of the patients believed that yoga and exercise are good self-management strategies. Responses like “I walk to feel less out of breath” (patient 1, male, 64 years old, COPD) and “I wake up at 4:00 a.m. and exercise for 1 hour. I perform exercises of the lower back, bending exercises, and breathing exercises” (patient 10, male, 69 years old, COPD) were common. Some patients were aware of the lifelong dependence on treatment and had started practicing yoga and exercising independently. One patient who regularly exercised was convinced that there is no alternative to it. He said:

In this disease of lungs, breathlessness, exercise is very important. Only that much, nothing else.

Patient 11, male, 48 years old, COPD

In contrast, he also believed that yoga would not work as a treatment regimen for patients with CRDs. He said:

Yoga is performed to strengthen the body and bones alone, and it is not needed in asthma-like conditions.

Patient 11, male, 48 years old, COPD

Gender-based differences emerged from patients’ narratives around their adherence to an exercise routine. Women frequently stated that household work restricts their ability to adhere to an exercise routine. One female patient said:

I like to do exercises, but I have time constraints. If I exercise in the morning, my son will go to the college without his tiffin.

Patient 7, female, 43 years old, asthma

Conversely, the accounts of male patients never included any household responsibility. A male patient said:

I don’t do any work; I only go for a walk. I walk for 2 km and then come back. Since last 10 years, I am doing this.

Patient 14, male, 67 years old, COPD

The patients who practiced yoga regularly had not learned the proper technique of yoga but performed a few yoga postures and breathing exercises, as advised by a friend or family member or as seen on television (televised programs on yoga) or in YouTube videos.

I do some exercises shown by Ramdev Baba (an Indian yoga guru). I exercise in the morning. I watch it on TV. It’s there every day, early morning from 5:00 a.m. to 6:00 a.m. Whatever I can, I do.

Patient 2, male, 70 years old, COPD

We have a union of retired people. We gather and have meeting every month. They suggested to do pranayama (breathing exercises) to feel better.

Patient 5, male, 79 years old, asthma

Many patients developed strategies based on their experiences of symptomatic relief. The patients reported several changes in their food habits, including overall food intake, nutritional value of the food, meal timings, and restrictions on certain food items. Alterations in food habits included the consumption of nonfried, nonspicy homemade food.

I cannot eat fried snacks like bhaji (fritters). I become breathless, and my cough increases.

Patient 4, male, 79 years old, COPD

In addition, patients mentioned consuming medicinal plant–based decoctions to get relief from the symptoms of cough and cold.

I consume a decoction, a kadha, with ginger, black pepper. I consume it when it’s very hot. I feel good.

Patient 1, male, 64 years old, COPD

#### Theme 2: Perceptions of PR

The second theme highlighted the knowledge of patients and medical professionals about PR, the perceived barriers to PR, and the perceptions of the digital mode of PR delivery.

##### Subtheme 2.1: Knowledge of PR

None of the patients identified with the term “pulmonary rehabilitation,” but many were aware of the exercise component and the benefits.

If I continue with the exercises, then I don’t get any trouble, but if I don’t do the exercises, then I get chest pain, feel breathless. Everybody needs exercise to remain healthy.

Patient 12, female, 44 years old, COPD

With medicines, we get immediate effect, but if we exercise for 1 hour every day, it will be more beneficial than the tablets. It will benefit us for a longer time.

Patient 9, male, 58 years old, COPD

The knowledge of the medical professionals about PR varied a lot depending on their experience. Physiotherapists not specialized in cardiopulmonary rehabilitation also treated patients with CRDs. One doctor (chest physician) and one cardiopulmonary physiotherapist who were experienced in referring and treating patients with PR had detailed knowledge of what PR constitutes:

Only breathing exercises are not sufficient to treat COPD, asthma patient, because strengthening of the muscles is also needed for them, and it is the major part of pulmonary rehabilitation.

Physiotherapist 3, female

Other medical professionals were aware of some of the breathing exercises to be prescribed to patients with CRDs. One of the doctors mentioned:

I tell them (the patients) to do deep-breathing exercises, deep inhalation, and a daily walk of 30 minutes. No hard exercises by which they will feel tired or exhausted.

Doctor 3, male

Another physiotherapist referred to PR as “chest physiotherapy.”

As the first point of professional contact for patients, doctors have the ability to convince them to undergo PR by properly knowing about the condition and its treatment. One of the physiotherapists pointed out:

If the doctors ask the patients to opt for PR, they would definitely opt for [PR].

Physiotherapist 1, male

##### Subtheme 2.2: Barriers to PR

In addition to the physical and social limitations on activity cited in theme 1, patients listed certain challenges to regularly exercising.

I can do some physical exercises and breathing exercises too. I won’t be able to jump and all.

Patient 2, male, 70 years old, COPD

According to all 9 medical professionals, PR plays an equal role to pharmacological treatment for patients with CRDs.

PR is necessary for these patients, and it is much useful. They need to continue their medicines but keep doing the strength exercises too.

Physiotherapist 1, male

CRD, especially COPD, is a progressive disease. If the patients are not on PR, they would end up frequently in the hospital with exacerbations. There is a need to complement the medication with strengthening of their lungs. That happens through PR.

Doctor 3, male

However, the lack of PR services was highlighted as an issue by some of them. One of the doctors expressed the need for personnel and infrastructure by saying:

There should be a big hall, it should be easily accessible, everything should be available with some sort of emergency backup. By chance the patients feel the need for oxygen, nebulization, some IV access, one sister. It should be a good rehab center.

Doctor 2, male

Other doctors mentioned:

As infrastructure, what PR requires is a separate room, but even that is not made available. Contrary to the popular belief, not many instruments are required for PR. Orthopedic and other branches require more instruments. Space and a dedicated physiotherapist who can teach is what we need.

Doctor 3, male

PR is crucially important for these patients. In fact, I have always wanted to have a proper PR setup for my patients. We lack in providing that.

Doctor 4, female

I don’t know any fully functional PR setups in the city except for one.

Physiotherapist 2, female

The medical professionals’ views on affordability of PR services on a regular basis varied between “not being a major hurdle for the patients” (doctor 2, male) to “posing heavy economic burdens on the families” (physiotherapist 3, female). Most of them were not sure about patients adhering to a long treatment program like PR because of the lack of awareness about the why and how of PR.

There is a need for PR. Patients don’t perceive that actually. There is no awareness. So the patient has no idea of that.

Doctor 1, female

### COVID-19 Pandemic

Although the COVID-19 pandemic had a devastating effect on all aspects of people’s lives, none of the patients mentioned that they faced any additional difficulties accessing treatment for their CRDs during the pandemic. The medical professionals mentioned that although they had feared worse outcomes for patients with COPD and asthma during the pandemic, these patients did not face any more difficulties than the general population. According to one of the doctors, this could be because of “the beneficial effect of the inhaled corticosteroids they were taking as a routine treatment” or the fact that “they were overprotective, overcautious, and did not leave home at all, because they were already worried about their lung health” (doctor 3, male).

#### Subtheme 2.3: Digital Mode of PR

Many patients had not used a smartphone before. Their responses included:

I don’t know anything about the mobile phone. Don’t know which button is where. I’ll take help from my son to show it (the exercise videos) to me.

Patient 1, male, 64 years old, COPD

I don’t have a smartphone. I can’t see videos on it.

Patient 7, female, 43 years old, asthma

Patients without an education and some patients with low education levels could not read text, including in the local language. Although most patients were naive to smartphone technology or had difficulty in reading, they were enthusiastic about enrolling in an application-based digital PR program. For them, the digital mode was an avenue to save the time and money needed to travel to the hospital.

An app will reduce the to and from home to hospital. If you show me the exercises on phone, I will be able to do it. Once I watch it, I will be able to do it.

Patient 1, male, 64 years old, COPD

They were confident about managing the exercises with the help of family members, once they were properly oriented.

If you tell the details of an app to my son, he will understand all these things, and how to operate this, and then he will explain me.

Patient 9, male, 58 years old, COPD

In addition to exercise videos and instructions, one of the participants asked to “add information about the exercises and its related benefits” as this information would “keep me motivated” to exercise regularly as per the instructions (patient 9, male, 58 years old, COPD).

Although some of the patients were unsure about whether they would be able to access the information via an app, many realized its importance in making changes to the program.

I will certainly do the exercises and will send the required information. I will send all necessary information to you from my side.

Patient 11, male, 48 years old, COPD

Although many patients were ready and confident about using the technology and exercising by watching on an app, the medical professionals were reluctant to depend on a PR program delivered fully through an online mode. Human contact was thought to be essential.

Although the patients get a lot of care and support from the family, they need a professional helping hand. That makes a lot of difference. They want that touch. When we help them get their dumbbells up and down or when we actually put their leg on treadmill, that helps them.

Doctor 2, male

The doctor further recommended a hybrid mode of delivery, stating:

After 2-3 sessions, there can also be a follow-up visit, so that time, you can see and you can ask the patients how they are doing.

Doctor 2, male

In addition, the medical professionals were concerned about the longer duration required for a digital program and the continuity of patients’ interest and the way the exercises would be performed. According to the medical professionals, social factors would largely determine the success of a digital intervention. They felt that patients residing in urban areas and patients with an education who have been exposed to smartphones before would be better candidates for a digital PR. Furthermore, internet connectivity would be a crucial factor.

It (digital mode of PR) will depend on the type of patient. Literate and educated class may follow it nicely. We will need to worry about the rural people or illiterate people who may not follow it.

Doctor 1, female

A doctor with experience of running a PR program for patients with CRDs mentioned the need for specific equipment to exercise with (eg, dumbbells to use while exercising).

People normally want some gadgets. If you just ask them to do just home exercises, then they are not convinced about PR. Looking at the equipment, the patients used to feel better. It is something that they wanted to do and have never done it before. So, these gadgets definitely attract them.

Doctor 2, male

For the digital mode of delivery, all the medical professionals recommended that patients be supported by their family to use technology, considering the age of most of the patients with CRDs. The physical interaction was seen as important to keep up their morale.

At the beginning, COPD patients become breathless with some exercises. They get demotivated because of this. In the absence of a practitioner, it becomes difficult to keep their morale up.

Doctor 3, male

## Discussion

### Principal Findings

This research indicates a clear demand from patients for PR to feel better and to reduce the feeling of helplessness that arises due to their dependency on their family members and inhalers. They are inclined to manage CRDs by getting symptomatic relief through medicines and inhalers. The patients’ knowledge of how to use their inhalers and medicines is poor, however. Some of them manage their condition through exercises and lifestyle modifications, but the knowledge of the what and why of the exercises is limited and not guided by professionals. The interviewed medical professionals highlighted the huge and ever-increasing need for PR but were also aware of the limitations of the available infrastructure and expertise to deliver such services.

The research also highlighted the need for educating medical professionals about the specifics of PR and its benefits to patients. The medical professionals showed a willingness for referrals, but their knowledge of exercises, the benefits of PR, and its components was limited. This is especially the case for those who are not trained fully to treat patients with CRDs but cater to the needs of patients because there is an unmet demand for cardiopulmonary specialists [26]. A low knowledge of a structured PR program among medical professionals as well as patients’ dependence on their treating doctors for referrals and adherence to PR have been observed in a study among medical practitioners in Australia [27]. Another study with general practitioners in Denmark observed that medical professionals mainly provide pharmacological treatment, whereas PR is left to the patients to explore directly with health centers, creating a disconnect and contributing to poor PR uptake by patients [11]. The improved knowledge of medical professionals would “sell” PR better to patients who would benefit from participating.

The poor knowledge of patients, especially about the proper use of inhalers and medications, was highlighted in this study. The results indicated the potential overreliance on inhalers and medications for some patients and a lack of knowledge of the proper use, leading to an inability to feel better, even with excessive use. The education component of the PR program thus becomes crucial for key challenges, such as optimizing the inhaler technique.

The idea of a home-based digital PR program appealed to the patients in this research. However, the medical professionals were cautious about the success of a digital PR program in India and advocated for a hybrid mode. For a low-resource setting, such as India, a completely digital program has proven to be a challenge, given the reluctance and hesitance in using a smartphone. In addition, poor health literacy, a lack of technology and resources, and a lack of expertise were found to be barriers to digital PR [28]. However, because of the COVID-19 pandemic, people now seem more receptive to the idea of a home-based [29] or digital [20] intervention. Telerehabilitation was suggested for the management of COPD in India in a recent study [21]. This study suggests that a home-based or hybrid model of PR could improve access to PR services in India as it will reduce the need for travel, specialist equipment, and setup, which have been reported as barriers in previous studies [9,16]. However, further evidence through clinical trials would help establish the digital mode of PR as a treatment strategy for CRDs.

For a PR program to be successful, it needs to be culturally appropriate. Adaptations, including incorporating national dances and psychological support for stigma, have been documented in a qualitative study from Kyrgyzstan as part of developing a PR program [30]. In the Indian context, yoga may offer an equivalent culturally acceptable adaptation to complement core PR components [31]. There was a strong desire for the use of yoga in the management of CRDs by the patients in this research. Yoga-based remotely delivered rehabilitation has been reported to be an effective alternative to PR [32]. Although none of the medical professionals have referred their patients for yoga, when asked about the usefulness of yoga in the management of CRDs, they responded positively.

### Limitations

This study was limited to 1 geographical area (Pune City) and used a convenience sampling method. Considering the size of the country, the findings of this study cannot be generalized to India as a whole. Further investigation in different parts of the country may elicit different responses, challenges, and opportunities. However, our insights provide a strong foundation for future research to build upon, given that PR is in its relative infancy in India. Patients with other CRDs may have different perceptions, experiences, needs, and challenges than those mentioned in this study. This study would have benefitted from obtaining the patients’ family members’ perspective on PR, who would likely play an important part in PR uptake, adherence, and completion.

### Conclusion

Individuals with CRDs in India currently manage their disease with nonguided strategies but are eager to improve and would benefit from a guided PR program to feel better. In the Indian context, including yoga in a PR program could be a way of making PR culturally congruent and more successful. A home-based PR program, with delivery facilitated by digital solutions, would be welcomed by patients and the health care professionals involved in their care. However, health care professionals have concerns over low digital literacy, low resource availability, and a lack of expertise. The digital PR intervention should be flexible to individual patient needs and should be complemented with human contact and a feedback mechanism for both medical practitioners as well as patients for better uptake.

## Appendix

Multimedia Appendix 1 In-depth interview guide for patients.

Multimedia Appendix 2 In-depth interview guide for medical professionals.

## Abbreviations

COPD: chronic obstructive pulmonary disease
CRD: chronic respiratory disease
LMIC: low- and middle-income country
NIHR: National Institute for Health Research
PR: pulmonary rehabilitation

## Data Availability

The data of the study are available from the corresponding author upon reasonable request.
