# Supplementary material for: Perspectives of Patients With Chronic Respiratory Diseases and Medical Professionals on Pulmonary Rehabilitation in Pune, India: Qualitative Analysis
Source: JMIR Form Res. 2023 Nov 7;7:e45624. doi: 10.2196/45624 (PMC10664007; doi:10.2196/45624)
Supplement: Multimedia Appendix 1 [file formative_v7i1e45624_app1.docx]

**The needs, preferences and challenges for developing Pulmonary Rehabilitation: Qualitative insights among various stakeholders**

**In depth interview Guide: Patients**

Interviewer: Hello, my name is [insert]. I would like to ask you some questions about pulmonary rehabilitation for people living with COPD. Please note that this interview is being recorded. We would like to hear your views on pulmonary rehabilitation and how it could be adapted in India.

1. **What is your experience of living with COPD?**

(PROMPTS: pre-COVID/ medications/ employment).

Probing questions:

- What would you list as the most significant thing about this condition? In what ways? Give examples. (Prompts: personally/ professionally; impact on your life/ activities in general etc.)

1. **What do you do to manage your condition/symptoms?**

(PROMPTS: pre-COVID/ Medications)

Probing questions:

- PROMPT COVID: What has changed due to COVID? During COVID?
- PROMPT COVID: Do you think you will return to previous strategies or will you change strategies due to COVID? How might these changes affect you in the future?
- Do you get any support from anyone (family/ friends/ neighbors/ colleagues) in managing the condition? If yes, who? And How? Do they find or share any disease management strategies or ways with you?

1. **Everyday activities**

- When are you most active in the day?
- PROMPT COVID: Has this changed due to COVID/ during COVID? How so?
- What physical activities (PROMPTS: pranayama, yoga, physical exercises) would you be happy doing more of? What physical activities (PROMPT: pranayama, yoga, physical exercises) would you not be happy doing more of?
- Would you like to include any activities in your regular exercise routine? If so, what activities would you like to include?

**Interviewer: Provide brief overview of PR**

1. **Mode of delivery of PR (Digital or remote PR feasibility/ opinions)**

Probing questions:

- What are your initial thoughts about digital PR? Why?
- Do they have any health/exercise apps? Do you currently have any health/ exercise related apps that help remind you to do certain things? Like a diary reminder or alert?
  - If so, how do you feel about using them?
  - What’s helpful/ not helpful about them?
  - How could they work better?
- Do you regularly use a smartphone that belongs to either yourself or a friend/family member?
  - If so, what do you use it for?
  - Why do you use the particular apps (mentioned by the respondents)?
- What advantages do you think there are in using a smartphone app to manage your health? More specifically, COPD? How would you hope digital PR might help you?
- What challenges/ concerns do you think there are in using a smartphone app to manage your health? More specifically, COPD?
- Would you use an app to manage your COPD through PR? What would you like to have in the app? (Probe: what components? Features? What material (audio/ visual/ text/ photo instructions?))

What would you expect to happen on the digital PR program?

- What do you think should be included in digital PR? Why? (PROMPTS: education, information, types of exercises (pranayama/ yoga))
- If you were to be asked to do yoga as part of digital PR, what would you think? Why? What do you think the challenges might be with yoga as part of digital PR? For people with COPD?
- Are there any circumstances you would have to change in order to take part in digital PR in the future? (PROMPTS: Family/friends support, device internet/accessibility etc.)
- What would be your thoughts on digital PR without the doctor or physio?
