# Supplementary material for: Perspectives of Patients With Chronic Respiratory Diseases and Medical Professionals on Pulmonary Rehabilitation in Pune, India: Qualitative Analysis
Source: JMIR Form Res. 2023 Nov 7;7:e45624. doi: 10.2196/45624 (PMC10664007; doi:10.2196/45624)
Supplement: Multimedia Appendix 2 [file formative_v7i1e45624_app2.docx]

**Pulmonary Rehabilitation related perceptions, practices among various stakeholders**

**Medical professionals (Doctors & Physiotherapists) Interview Schedule**

Interviewer: Hello, my name is [insert]. I would like to ask you some questions about pulmonary rehabilitation for COPD patients. Please note this interview is being recorded. We would like to hear your views on pulmonary rehabilitation and how it could be adapted in India.

1. **FOR DOCTOR & PHYSIO: Tell us about your experience treating COPD patients?**

Probing questions:

- PROMPT COVID: Has this changed due to COVID/ during COVID? How so?

1. **Ask DOCTOR: How would you describe PR to patients?**

Probing questions:

- How do you refer patients to PR? Can you tell us about the referral pathway(s)?
- What would be your reasons for referring patients to PR?
- Do you think there will be any challenges referring patients to PR?
- How do you think these could be resolved?

1. **Ask DOCTOR & PHYSIO: How important do you think it is to provide PR for COPD patients?**

**Why?**

Probing questions:

- How does this compare to other forms of treatment such as inhalers, medication, oxygen?

1. **Ask DOCTOR: How do you think patients would respond to your referral for PR?**
2. **Ask DOCTOR & PHYSIO: How do you think patients would get on with PR?**

Probing questions:

- Do you think there will be any challenges for patients taking part in PR?
- How do you think these can be resolved?
- How should PR be delivered during COVID? (PROMPTS: home, community, digital, hospital). Why? Are there any challenges/ barriers associated with EITHER home/ community/ digital/ hospital PR? PROMPT: pre-COVID
- What adaptations do you recommend for the safe delivery of PR during the COVID pandemic? (if participant mentions group based setting, PROMPT: safety, handwashing, group based activities, social distancing, face masks)

1. **Ask DOCTOR & PHYSIO: What do you think should be included in PR? PROMPT: pre-COVID. Why? (PROMPT, education, information, types of exercises)**

Probing questions:

- Are the any activities or hobbies that could be useful for patients in PR? (PROMPT: yoga)
- If patients were asked to do yoga as part of PR, how do you think they would respond? Why?
- What do you think the challenges might be with yoga?
- How do you think these challenges can be resolved?

1. **Mode of delivery of PR (Digital or remote PR feasibility/ opinions)**

Probing questions:

- What do you think about digital PR? (PROMPT: Feasibility, Acceptability, COVID)
- Would you recommend using an app to manage COPD through PR for your patients?
- What advantages do you think there are in using digital PR for COPD? (especially during COVID)
- What challenges/ concerns do you think there are in using digital PR for COPD? (Prompts: Any ideas on how can digital PR be offered to eligible patients in the remote locations/ More visual aids/ more remote follow up from physios, doctors, etc)
- What do you think about yoga being part of digital PR? (PROMPT: Any ideas on ensuring proper technique, Safety etc.)
- How do you feel about patients doing digital PR without support from Doctors or Physios? Do you think there are any advantages? Any challenges/concerns?
